# Bifunctional dendrons for multiple carbohydrate presentation via carbonyl chemistry

**DOI:** 10.3762/bjoc.10.177

**Published:** 2014-07-25

**Authors:** Davide Bini, Francesco Nicotra, Laura Cipolla

**Affiliations:** 1 Department of Biotechnology and Biosciences, University of Milano-Bicocca, Piazza della Scienza 2, 20126 Milano, Italy

**Keywords:** bis-MPA, carbohydrates, dendrons, levulinic acid, multivalency, multivalent glycosystems

## Abstract

The synthesis of new dendrons of the generations 0, 1 and 2 with a double bond at the focal point and a carbonyl group at the termini has been carried out. The carbonyl group has been exploited for the multivalent conjugation to a sample saccharide by reductive amination and alkoxyamine conjugation.

## Introduction

Recognition processes between glycans and their receptors are of paramount relevance in several biological phenomena, both in physiological [1–2] and in pathological [3–5] conditions. These processes can be exploited in diagnostic tools [6–7], in nanobiotechnology applications [8], and in the development of smart biomaterials for regenerative medicine [9–12]. Beside the variation of carbohydrate residues in glycoconjugates, a key issue in the recognition process is their spatial topographical presentation eliciting high affinity recognition events. In order to better understand these phenomena, dendrimers and dendrons have been developed to provide multivalent glycoconjugates [13–14]. Here, we propose the synthesis of novel dendron structures which allow for the multivalent conjugation of carbohydrates via carbonyl chemistry.

## Results and Discussion

**Figure 1:**
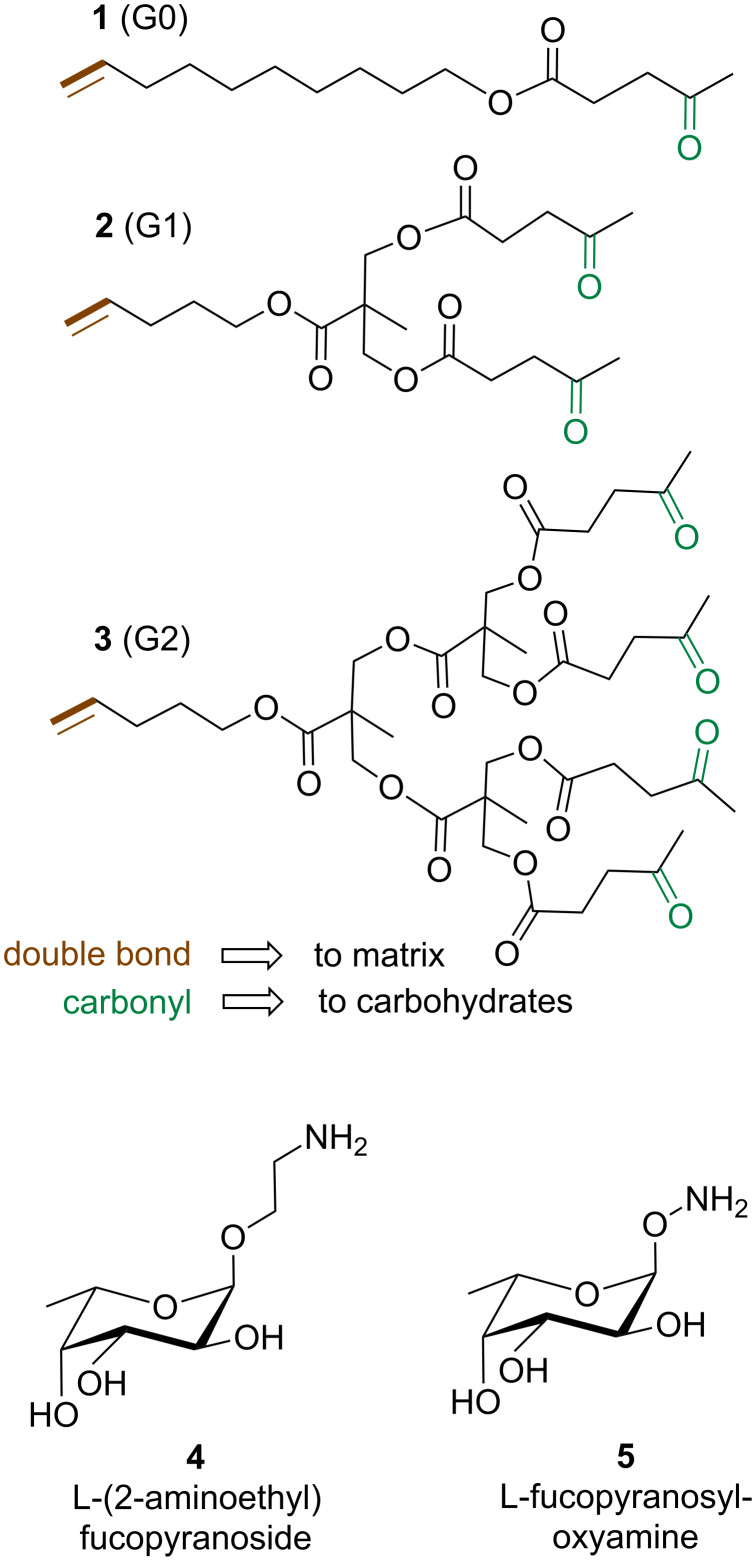
Synthesized G0, G1 and G2 dendrons and functionalized saccharides used for carbonyl conjugation.

**Scheme 1:**
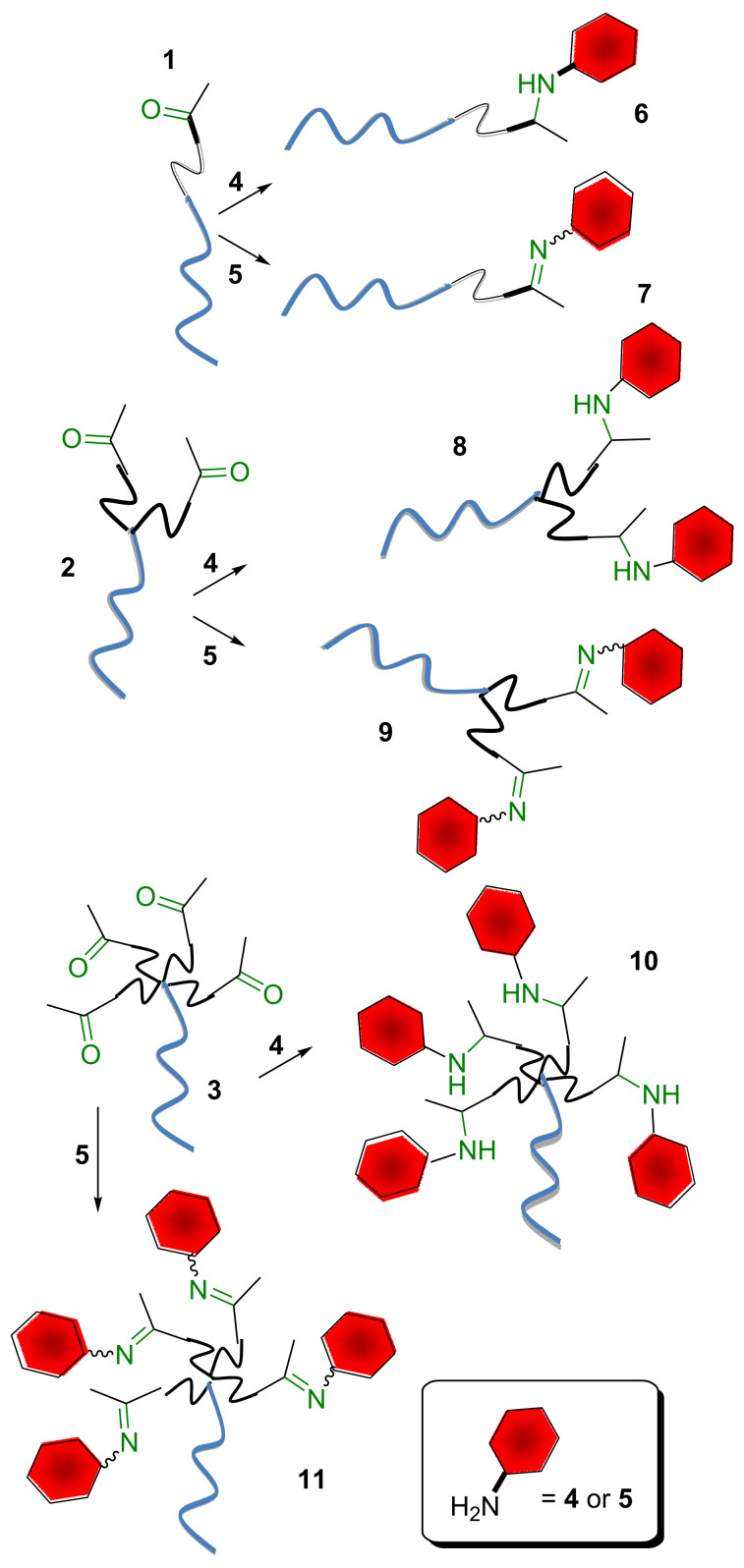
Schematic depiction of dendron conjugation to saccharides by carbonyl chemistry.

The heterobifunctional dendrons were designed in order to have bio-orthogonal functional groups at the focal point and at their termini. More specifically, a double bond was placed at the desired matrix as the focal point for further conjugation by thiol–ene chemistry, and carbonyl groups were added at the termini. The carbonyl groups can be exploited for carbohydrate functionalization [15–16] by reductive amination, oxime or hydrazone formation to yield suitably functionalized saccharides (Figure 1). Given the relevance of L-fucose in mammal oligosaccharides, α-L-(2-aminoethyl) fucoside [17] and α-*O*-L-fucopyranosyloxyamine [18] were used as sample monosaccharides for the conjugation of the dendron (Scheme 1).

## Synthesis of dendrons

**Scheme 2:**
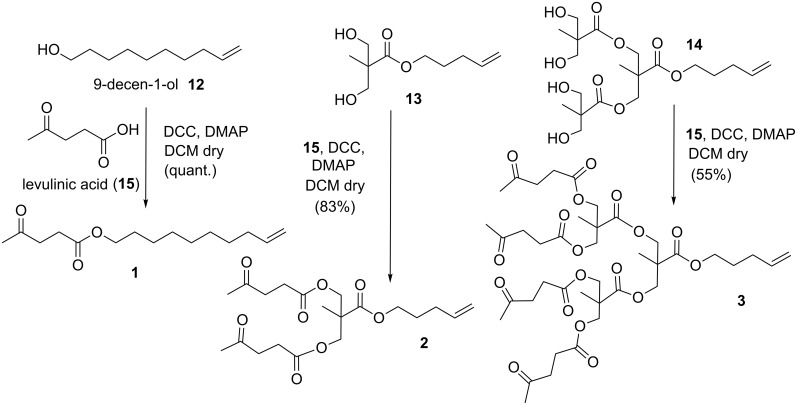
Synthesis of the dendrons.

Zero, first and second generation heterobifunctional dendrons **1–3** were synthesized starting from 9-decen-1-ol (**12**) or selected building blocks **13** and **14** (Scheme 2) [11] by esterification reactions with levulinic acid (**15**). Building blocks **13** and **14** were synthesized starting from bis-(hydroxymethyl)propionic acid (bis-MPA) and bromo-1-pentene [11] in one and four steps, respectively.

## L-Fucose derivatives synthesis

α-L-(2-Aminoethyl) fucopyranoside (**4**) and α-O-L-fucopyranosyloxyamine (**5**) were synthesized from commercial L-fucopyranose in 4 and 5 steps, respectively, as already reported by Flitsch and co-workers [17] and Dumy and co-workers [18].

### Dendron conjugation to L-fucose by reductive amination

**Scheme 3:**
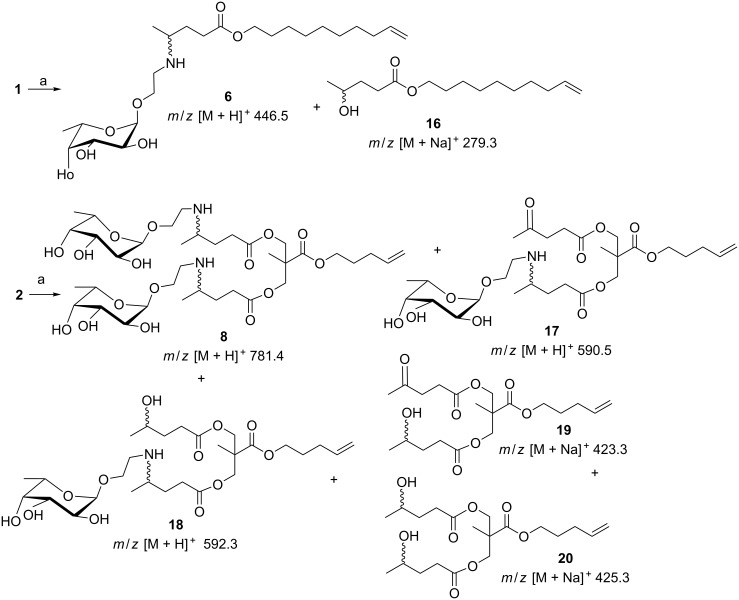
Dendron conjugation to fucose moieties by reductive amination. Reagents and conditions: a) **4**, 3 M Na_2_SO_4_, AcOH, NaCNBH_3_, EtOH, 80 °C, 6 h.

α-L-(2-Aminoethyl) fucopyranoside (**4**) was conjugated first to G0 dendron **1** by reductive amination in the presence of NaCNBH_3_ (Scheme 3). The reaction afforded the desired glycosylated dendron **6** in 27% yield. The very low yield was ascribed to the competing carbonyl reduction to the corresponding alcohol **16** as a byproduct.

The same reaction on the G1 dendron **2** gave an even more complex mixture of products, identified by mass spectrometry (System Applied Biosystems MDS SCIEX instruments: Q TRAP, LC/MS/MS, turbo ion spray and Q STAR elite nano spray) performed directly on the TLC spots following literature procedures [19]. By mass values, the mixture was composed of the desired fucosylated dendron **8** as the minor product together with the monoglycosylated derivatives **17** and **18** and the alcohols **19** and **20**. In order to reduce the formation of alcohol byproducts, a “milder” reducing agent such as Na(AcO)_3_BH was tried, but without any success.

**Scheme 4:**
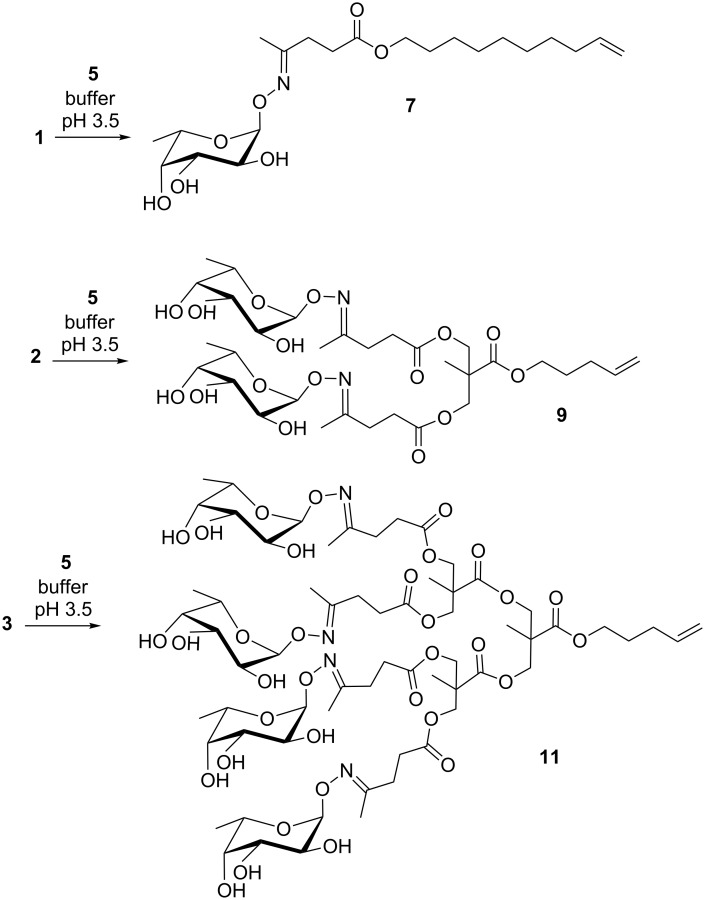
Dendron conjugation to fucose via oxime ligation (buffer = citrate buffer).

Given the high extent of byproducts and the low efficiency of the glycoconjugation to the G0 dendron and G1 dendron by reductive amination, we decided to evaluate the possibility to obtain better conjugation yields by oxime ligation. Thus, G0, G1 and G2 dendrons **1**–**3** were reacted with α-O-L-fucopyranosyloxyamine (**5**) in citrate buffer at pH 3.5 [20] (Scheme 4). Due to the partial hydrophobic nature of the dendrons **1**–**3** they do not fully dissolve in the buffer, and the solution is not completely clear.

The dendrons **1**–**3** were reacted overnight at room temperature with α-O-L-fucopyranosyloxyamine (**5**) affording the desired glycoconjugate structures **7**, **9** and **11** in quantitative yields.

## Conclusion

In conclusion, novel G0, G1 and G2 dendrons suitable for glycoconjugation by carbonyl chemistry were synthesized. The conjugation of the saccharide by reductive amination was characterized by a low efficiency. On the other hand, the oxime ligation afforded the glycoconjugated dendrons in quantitative yields. The glycosylated dendrons can be exploited for further chemoselctive thiol–ene reactions with matrices suitably functionalized with thiol groups, i.e., cysteine residues in proteins.

## Experimental

### General methods

All chemicals were purchased from Sigma-Aldrich and used without further purification. All solvents were dried over molecular sieves, for at least 24 h prior to use, when required. When dry conditions were required, the reaction was performed under an Ar atmosphere. Thin-layer chromatography (TLC) was performed on silica gel 60 F254 coated glass plates (Merck) with UV detection when possible, or spots were visualized by charring with a conc. H_2_SO_4_/EtOH/H_2_O solution (10:45:45 v/v/v), or with a solution of (NH_4_)_6_Mo_7_O_24_ (21 g), Ce(SO_4_)_2_ (1 g), conc. H_2_SO_4_ (31 mL) in water (500 mL) and then by heating to 110 °C for 5 min. Flash column chromatography was performed on silica gel 230–400 mesh (Merck). Routine ^1^H and ^13^C NMR spectra were recorded on a Varian Mercury instrument at 400 MHz (^1^H) and 100.57 MHz (^13^C). Chemical shifts are reported in parts per million downfield from TMS as an internal standard, *J* values are given in Hz. Mass spectra were recorded on System Applied Biosystems MDS SCIEX instruments: Q TRAP, LC/MS/MS, turbo ion spray and Q STAR elite nanospray.

**General procedure for levulinic acid condensation (compounds 1–3):** To a 0.1 M solution of the appropriate compound dissolved in dry DCM, levulinic acid (1.2 equiv), DMAP (0.2 equiv) and DCC (1.5 equiv) were added, and the reaction mixture was stirred at room temperature (for 1 to 24 h, depending on the substrate). The precipitates were filtered off and washed with CH_2_Cl_2_. The solvent was evaporated, and the residue was purified by column chromatography on a silica gel column with a suitable eluent. See Supporting Information File 1 for full experimental data.

**General procedure for reductive amination reaction (compounds 6, 8)**:The appropriate dendron (1 equiv) and α-L-(2-aminoethyl) fucoside (**4**, 1 equiv) were dissolved in EtOH (0.1 M in respect to **4**). AcOH (1 equiv) and 3 M Na_2_SO_4_ (1% of solvent volume) were added, and the mixture was heated under reflux for 2 h. NaCNBH_3_ (1.5 equiv) was then added, and the reaction was heated under reflux for further 4 h. See Supporting Information File 1 for full experimental data.

**General procedure for dendron/ alkoxyamine conjugation (compounds 7, 9, 11):** The appropriate dendron (1 equiv) and α-O-L-fucopyranosyloxyamine (**5**, 1 equiv) were dissolved in citrate buffer (pH 3.5, 0.1 M in respect to **5**) and stirred at room temperature overnight. The mixture was concentrated and the product isolated. See Supporting Information File 1 for full experimental data.

## Supporting Information

File 1 Experimental part.
