# Supplementary material for: Bifunctional dendrons for multiple carbohydrate presentation via carbonyl chemistry
Source: Beilstein J Org Chem. 2014 Jul 25;10:1686–91. doi: 10.3762/bjoc.10.177 (PMC4143090; doi:10.3762/bjoc.10.177)
Supplement: File 1 — Experimental part. [file Beilstein_J_Org_Chem-10-1686-s001.pdf]

Supporting Information

for

Bifunctional dendrons for multiple carbohydrate presentation via carbonyl chemistry

Davide Bini, Francesco Nicotra and Laura Cipolla*

Address: Department of Biotechnology and Biosciences, University of Milano-Bicocca, Piazza della Scienza 2, 20126 Milano, Italy

Email: Laura Cipolla - laura.cipolla@unimib.it

*Corresponding author

Experimental part

Dendron 1: Flash column chromatography (petroleum ether/EtOAc, 85:15). ^{1}H NMR (400 MHz, $CDCl_3$) δ 5.72 (ddt, *J* = 16.9, 10.2, 6.7 Hz, 1H, C*H*=CH_2), 4.95 – 4.79 (m, 2H, CH=CH_2), 3.98 (t, *J* = 6.8 Hz, 2H, CH_2O), 2.67 (t, *J* = 6.5 Hz, 2H, CH_3COCH_2), 2.49 (t, *J* = 6.5 Hz, 2H, $CH_2CH_2CO_2$), 2.11 (s, 3H, , CH_3CO), 1.96 (q, *J* = 7.1 Hz, 2H, CH_2-CH=CH_2), 1.58 – 1.48 (m, 2H, O-CH_2-CH_2), 1.35 – 1.17 (m, 10H, CH_2) ppm; ^{13}C NMR (100.57 MHz, $CDCl_3$) δ 206.52, 172.69 (C=O), 138.99 (*C*H=CH_2), 114.09 (CH=*C*H_2), 64.68 (*C*H_2O), 37.84 (CH_3COCH_2), 33.70 (*C*H_2-CH=CH_2), 29.77 (*C*H_3CO), 29.26, 29.10, 28.94, 28.79, 28.48, 27.88, 25.78 (CH_2) ppm; MS (TOF) *m/z*: 277.2 $[M + Na]^+$; found 277.1.

Dendron 2: Flash column chromatography (petroleum ether/EtOAc, 55:45). ^{1}H NMR (400 MHz, $CDCl_3$) δ 5.81 – 5.71 (m, 1H, C*H*=CH_2), 5.05 – 4.92 (m, 2H, CH=CH_2), 4.24 – 4.15 (m, 4H, CH_2O-CO-CH_2), 4.11 (t, *J* = 6.4 Hz, 2H, CH_2-CH_2-O-C=O), 2.71 (t, *J* = 6.4 Hz, 4H, CH_3COCH_2), 2.53 (t, *J* = 6.4 Hz, 4H, CH_2C$H_2$$CO_2$), 2.15 (s, 6H, CH_3CO), 2.08 (q, *J* = 7.0 Hz, 2H, CH_2-CH=CH_2), 1.76 – 1.66 (m, 2H, O-CH_2-CH_2), 1.21 (s, 3H, CH_3) ppm; ^{13}C NMR (100.57 MHz, $CDCl_3$) δ 206.35, 172.67, 172.16 (C=O), 137.19 (*C*H=CH_2), 115.43 (CH=*C*H_2), 65.41 (C-*C*H_2O), 64.53 (CH_2*C*H_2OCO), 46.22 (*C*-CH_2O), 37.79 (CH_3CO*C*H_2), 29.88 (*C*H_2-CH=CH_2), 29.75 (*C*H_3CO), 27.74, 27.59 (CH_3COCH_2*C*H_2, O-CH_2-*C*H_2), 17.78 (CH_3) ppm; MS (TOF) *m/z*: 421.2 $[M + Na]^+$; found 421.4.

Dendron 3: Flash column chromatography (petroleum ether/EtOAc, 25:75). ^{1}H NMR (400 MHz, $CDCl_3$) δ 5.77 (ddt, *J* = 16.8, 10.2, 6.6 Hz, 1H, C*H*=CH_2), 5.06 – 4.93 (m, 2H, CH=CH_2), 4.27 – 4.05 (m, 14H, CH_2O), 2.72 (t, *J* = 6.5 Hz, 8H, CH_3COCH_2), 2.54 (t, *J* = 6.4 Hz, 8H, CH_2C$H_2$$CO_2$), 2.15 (s, 12H, CH_3CO), 2.10 (dd, *J* = 14.3, 7.2 Hz, 2H, CH_2-CH=CH_2), 1.78 – 1.67 (m, 2H, O-CH_2-CH_2), 1.23 (s, 9H, CH_3) ppm; ^{13}C NMR (100.57 MHz, $CDCl_3$) δ 206.41, 172.17, 172.01 (C=O), 137.17 (*C*H=CH_2), 115.48 (CH=*C*H_2), 65.66, 65.21 (C-*C*H_2O), 64.82 (CH_2*C*H_2OCO), 46.54, 46.34 (*C*-CH_2O), 37.79 (CH_3CO*C*H_2), 29.92 (*C*H_2-CH=CH_2), 29.75 (*C*H_3CO), 27.70, 27.59 (CH_3COCH_2*C*H_2, O-CH_2-*C*H_2), 17.72, 17.58 (CH_3) ppm; MS (TOF) *m/z*: 894.4 $[M + Na]^+$; found 894.4.

Glycodendron 6: The mixture obtained as described in the general procedure was concentrated and the residue was purified by flash column chromatography (i-PrOH/NH_3, 95:5). ^{1}H NMR (400 MHz, CD_3OD) δ 5.80 (ddt, *J* = 16.9, 10.0, 6.7 Hz, 1H, C*H*=CH_2), 5.02 – 4.90 (m, 2H, CH=CH_2), 4.77 (bs, 1H, H-1), 4.07 (t, *J* = 6.6 Hz, 2H, CH_2OC=O), 3.94 (dd, *J*

= 13.2, 6.5 Hz, 1H, H-5), 3.85 – 3.79 (m, 1H, OCH_2CH_2NH), 3.77– 3.62 (m, 3H, H-2, H-3, H-4), 3.56 – 3.46 (m, 1H, OCH_2CH_2NH), 2.92 – 2.83 (m, 2H, CH_2NH), 2.78 (dd, *J* = 12.0, 6.5 Hz, 1H, CH_2CHCH_3), 2.40 (dd, *J* = 15.7, 8.7 Hz, 2H, CH_2C=O), 2.04 (dd, *J* = 15.4, 8.6 Hz, 2H, CH_2-CH=CH_2), 1.66 – 1.59 (m, 4H, $CH_2CH_2CO_2$, O-CH_2-CH_2- CH_2), 1.45 – 1.24 (m, 10H, CH_2), 1.21 (d, *J* = 6.6 Hz, 3H, CH_3-6), 1.12 (d, *J* = 6.3 Hz, 3H, CH_3-CH-NH) ppm; MS (TOF) *m/z*: 446.3 $[M + H]^+$; found 446.5.

(*E*/*Z*)-Gglycodendron 7: ^{1}H NMR (400 MHz, D_2O, major isomer) δ 5.61 (dt, *J* = 17.4, 7.2 Hz, 1H, CH=CH_2), 5.26 (bs, 1H, H-1), 4.87 – 4.71 (m, 2H, CH=CH_2), 3.95 – 3.85 (m, 2H, CH_2O), 3.82 – 3.69 (m, 3H, H-2, H-4, H-5), 3.65 (bs, 1H, H-3) 2.49 – 2.35 (m, 4H, CH_2C=O, CH_2C=N), 1.90 – 1.84 (m, 2H, CH_2-CH=CH_2), 1.78 (s, 3H, CH_3C=N), 1.50 – 1.43 (m, 2H, O-CH_2-CH_2), 1.24 – 1.12 (m, 10H, CH_2), 1.01 (d, *J* = 6.1 Hz, 3H, CH_3-6) ppm; MS (TOF) *m/z*: 438.2 $[M + Na]^+$; found 438.2.

(*E*/*Z*,*E*/*Z*)-Glycodendron 9: ^{1}H NMR (400 MHz, D_2O, major isomer) δ 5.74 (dt, *J* = 16.4, 6.8 Hz, 1H, CH=CH_2), 5.26 (bs, 2H, H-1), 4.94 – 4.87 (m, 2H, CH=CH_2), 4.19 – 4.10 (m, 4H, CH_2O), 4.04 (t, *J* = 5.8 Hz, 2H, CH_2CH_2O), 3.84 (q, *J* = 6.2 Hz, 2H, H-5), 3.79 – 3.71 (m, 4H, H-2, H-4), 3.67 (bs, 2H, H-3), 2.56 – 2.35 (m, 8H, CH_2C=O, CH_2C=N), 1.99 (dd, *J* = 13.7, 6.7 Hz, 2H, CH_2-CH=CH_2), 1.80 (s, 6H, CH_3C=N), 1.61 (dt, *J* = 13.3, 6.8 Hz, 2H, O-CH_2-CH_2), 1.13 (s, 3H, CH_3), 1.03 (d, *J* = 6.5 Hz, 6H, CH_3-6) ppm; ^{13}C NMR (100.57 MHz, D_2O, major isomer) δ 174.98, 174.43, 174.34 (C=O), 161.95 (C=N), 138.05 (CH=CH_2), 115.04 (CH=CH_2), 99.68 (C-1), 71.68 (C-3), 69.60 (C-4), 67.44 (C-2), 66.90 (C-5), 66.06 (C-CH_2O), 65.44 (CH_2CH_2OCO), 46.34 (C-CH_2O), 30.12, 30.06 (CH_2-C=O, CH_2-C=N), 29.34 (CH_2-CH=CH_2), 26.83 (O-CH_2-CH_2), 16.78 (CH_3), 15.24 (CH_3-6), 13.93 (CH_3-C=N) ppm; MS (TOF) *m/z*: 743.3 $[M + Na]^+$; found 743.3.

(*E*/*Z*,*E*/*Z*,*E*/*Z*,*E*/*Z*)-Glycodendron 11: ^{1}H NMR (400 MHz, D_2O, major isomer) δ 5.73 (ddt, *J* = 17.1, 10.3, 6.6 Hz, 1H, C*H*=CH_2), 5.26 (d, *J* = 2.7 Hz, 4H, H-1), 5.01 – 4.83 (m, 2H, CH=CH_2), 4.24 – 4.07 (m, 12H, CH_2O), 4.03 (t, *J* = 6.2 Hz, 2H, CH_2CH_2O), 3.83 (dd, *J* = 12.1, 5.8 Hz, 4H, H-5), 3.79 – 3.71 (m, 8H, H-2, H-4), 3.67 (bs, 4H, H-3), 2.55 – 2.37 (m, 16H, CH_2C=O, CH_2C=N), 2.00 (dd, *J* = 13.9, 7.1 Hz, 2H, CH_2-CH=CH_2), 1.80 (s, 12H, CH_3C=N), 1.64 (dd, *J* = 13.6, 6.8 Hz, 2H, O-CH_2-CH_2), 1.16 (s, 3H, CH_3), 1.12 (s, 6H, CH_3), 1.03 (d, *J* = 6.7 Hz, 12H, CH_3-6) ppm; ^{13}C NMR (100.57 MHz, D_2O, major isomer) δ 174.35, 174.20, 173.72 (C=O), 161.68 (C=N), 137.94 (*C*H=CH_2), 115.19 (CH=*C*H_2), 99.78 (C-1), 71.71 (C-3), 69.67 (C-4), 67.49 (C-2), 66.91 (C-5), 66.08 (C-*C*H_2O), 65.51 (CH_2*C*H_2OCO), 46.42 (*C*-CH_2O), 30.17, 30.07 (*C*H_2-C=O, *C*H_2-C=N), 29.50 (*C*H_2-CH=CH_2), 26.94 (O-CH_2-*C*H_2), 16.86 (CH_3), 15.30 (CH_3-6), 14.00 (*C*H_3-C=N) ppm; MS (TOF) *m/z*: 758.3 $[M + 2Na]^{2+}$; found 758.1.
